# ‘Honouring the storyteller’: the potential of Playback Theatre in health policy and systems research

**DOI:** 10.1093/heapol/czaf038

**Published:** 2025-06-25

**Authors:** Meena Putturaj, Radhika Jain

**Affiliations:** Centre of Adivasi Health, Institute of Public Health, 3009, II-A Main, 17th Cross, Krishna Rajendra Rd, Banashankari Stage II, Bengaluru, Karnataka 560070, India; First Drop Change Foundation, B705, Brigade Metropolis, Garudacharpalya, Bengaluru, Karnataka 560048, India

**Keywords:** Playback Theatre, health policy and systems research, storytelling

## Abstract

Stories and storytelling stimulate inquiries in health policy, and initiate and become an integral part of policy dialogues. They can also be used as a health policy advocacy tool. Storytelling is a compelling way to engage with various actors in the health policy realm, co-creating knowledge and action in the social world of health systems. Playback Theatre (PT) is an improvisational form of theatre in which audience members share their life stories, which are then enacted on the spot by a group of citizen actors. Citizen actors are everyday people who are not necessarily professional performers but are trained in PT. PT's emphasis on emotional expression and representation allows individuals to deeply engage with the stories of others, leading to greater empathy and understanding across diverse social groups. If applied with a critical consciousness, we argue that PT methodology can illuminate health policy and systems research storytelling processes, given its ontological and epistemological alignment with social constructivism and its orientation towards values such as human dignity and social justice. In this article, we explore the possibilities and the limits of PT for storytelling in the field of Health Policy and Systems Research, as it emphasises stories as much as the storyteller.

Key messages

- Stories and storytelling play vital roles in shaping health policy regimes. Stories help us think, reflect, and find human connections and relations, which are crucial for public and policy engagement.
- Playback Theatre—a performance art-based methodology—when applied critically, creates a congenial atmosphere for people to express emotional and social realities, both artistically and democratically, through their real-life stories.
- However, it is important for practitioners and researchers to critically examine and be self-reflective of using Playback Theatre as a methodology in Health Policy and Systems Research (e.g. who is doing Playback Theatre for whom, how, where, and why).

## Introduction

The policy community is most likely to be influenced in making policy decisions by compelling stories that are concise and relevant rather than by contextually insensitive, complex statistical data (Jewell and Bero 2008). This is partly because, as human beings, we tend to adopt cognitive and emotional shortcuts to avoid, reduce, or navigate the burden of decision-making in our everyday lives (Desmond *et al.* 2013, Cairney and Kwiatkowski 2017). Given that public policy processes are inherently political and value-based and are minimally evidence-informed (Mwisongo *et al.* 2016, Gore and Parker 2019, Friel *et al.* 2021), Health Policy and Systems Research (HPSR) is emerging as a field that seeks to explore these complexities within health policy regimes by examining the dynamic interplay of political, economic, social, and institutional factors that shape how policies are formulated, implemented, and evaluated (Sheikh *et al.* 2011). HPSR scholars are increasingly advocating for the use of narratives and storytelling to appeal to the (political) senses of the policy community and to shape health policy regimes (Steiner 2005, Jones and McBeth 2010, Toolan 2001, Dahlstrom 2014, Epstein *et al.* 2014). Storytelling refers to the artistic expression of individual or collective lived experiences through varied verbal and non-verbal formats. Elements in storytelling, such as the personal touch and attention to detail, captivate the listener and make real-life events more meaningful to both the teller and the listener (Bruner 1990, 1991). Storytelling is a cogent way of engaging with various actors for the co-creation of knowledge and action in the social world (of health systems). As argued by Hooks (2000), storytelling nurtures passionate ‘politics’ for instigating social change because stories are memorable and persuasive. Stories and storytelling may stimulate inquiries in health policy, and initiate and become an integral part of policy dialogues. They can also be used as a health policy advocacy tool (Fadlallah *et al.* 2019). Therefore, storytelling is an indispensable competency for health policy and systems researchers and practitioners to enable positive change in the health policy environment.

Several tools, approaches, and mediums enable storytelling, including narrative action reflection workshops, qualitative, and digital techniques (Gubrium and Turner 2011, Lorenz 2015, Fiddian-Green and Gubrium 2021). Among the various media of storytelling, theatre, especially applied theatre (a performative art form), stands out. Unlike conventional theatre (which is scripted most often), applied theatre is typically known for blurring the boundaries between actors and spectators because of its ability to actively engage the audience in the stories performed on the stage (O’Connor and O’Connor 2009). Applied theatre forms such as street theatre, theatre of the oppressed, theatre for living, theatre for development, and legislative theatre are used to advance, support, develop, and resist policy agendas, and their implementation has caught the attention of scholars in public policy (Mullen and Freebody 2022). Applied theatre is widely used by public policy scholars across the world as a methodological and theoretical approach to examine the relationship between policy and practice, because of its value in terms of politics, pedagogy, aesthetics, democracy, and ethics (Hallward 2006, Nisker *et al.* 2006, Rancière 2006a, 2006b, 2009). It is increasingly used to deepen our understanding and action on structural oppression as it offers safe/brave spaces for people to share their personal stories and their vulnerabilities that are often systematically silenced and thus go unrecognised in policy realms (Boal 1979, 1995, Thompson 2003, Nicholson 2005, Prentki and Preston 2009). Evidence demonstrates that politically framed applied theatre-based research is impactful in exploring the struggles of individuals and communities to secure respect and autonomy in circumstances of poverty, violence, and crime (Vuuren *et al.* 2021). However, in the HPSR field, scholars have not fully explored the potential of applied theatre and its various forms for storytelling. While there is growing evidence on the use of arts-based methodologies in health research, such as visual arts (e.g. photovoice, rich pictures), performance arts (e.g. street theatre, role play, drama), and literary arts (e.g. poetry) for knowledge production and translation (Fraser and Sayah 2011), the application of applied theatre forms like Playback Theatre (PT), Theatre of the Oppressed, Theatre for Living, and Legislative Theatre remains scarce within HPSR projects. Among these, PT, with its unique emphasis on personal storytelling and empathetic witnessing, offers critical value to HPSR, where the need to understand lived experiences is increasingly recognised as central to building equitable and people-centred health systems. PT is distinct from other participatory approaches and applied theatre methods because it centres directly on personal storytelling and spontaneous performance rather than pre-scripted or issue-driven material. Unlike other participatory approaches that often focus on problem-solving or structural critique, PT creates a non-confrontational, emotionally safe space where care-seeking individuals, health workers, policy actors, and communities in vulnerable circumstances can share their narratives without fear of judgment (Fox 1994, Salas 2013). This method aligns with the need in HPSR to bring rich experiential knowledge to the surface (Greenhalgh *et al.* 2016), revealing insights into trust, stigma, access barriers, and other systemic gaps that might otherwise be missed through more formal participatory frameworks. In this article, we explore the possibilities and limitations of PT as a storytelling method in HPSR, highlighting its dual emphasis on both the story and the storyteller.

## What is PT?

PT is an improvisational form of theatre in which audience members share stories from their lives and watch them enacted through music, dialogue and/or movement on the spot by a group of citizen actors. Citizen actors are everyday people who are not necessarily professional performers but are trained in the key aspects of PT, such as active listening, empathy, and improvisational storytelling (Fox 1994, Salas 2013). PT was developed by Jonathan Fox and Jo Salas in 1975. The origins of PT are marked by a departure from scripted theatre towards a return to an oral tradition of knowledge gathering, translation, and transmission. To support acts of collective remembrance within a culture of separation, PT grounds its practice in a theatre whose currency is not well-rehearsed prose but the emergent narratives that comprise the cultural knowledge of people (Sajnani and Johnson 2016). Fox and Salas intend PT to be an extension of an abandoned oral tradition within which communities generate insights regarding their lived experiences through the sharing and witnessing of each other's stories (Fox 1994, Salas 2013). The practice is simple and accessible, without elaborate scripts or props, allowing anyone to participate. As described by Salas (2011), PT can take place in two main formats: workshops and performances. In workshop settings, a trained facilitator guides a group, often composed of individuals who know each other, in sharing and acting out each other’s stories. These enactments hold personal and collective significance within the group. In contrast, performance settings involve a designated audience and a trained ensemble of actors, a conductor, and musicians. In both formats, the storytelling process fosters an embodied and imaginative dialogue (Salas 2011). Practitioners are expected to support one another, emphasising collaboration, solidarity, and ethical conduct. Initially, some support from trained practitioners is necessary for laypersons to begin performing PT. People who undergo training and continuously practice PT eventually become performers. According to Ng (2015), a PT practitioner, building citizen artistry in PT involves three elements: artistic condensation, where actors distil a story's essence through minimal, expressive forms; active ensemble practice to foster responsive group collaboration; and physical theatre techniques to communicate emotions and narratives through the body, beyond words. Once a community is exposed to PT, its members often continue practising it together. Through regular practice, they find healing, strengthen their bonds, and use PT as a tool to maintain community cohesion and promote collective well-being. For instance, Jeevika, an organization in Bengaluru, India, dedicated to the empowerment of bonded labourers, uses PT as a tool for communities to come together, share their challenges, and experience collective support against modern slavery (Jeevika 2025). PT thus serves as a powerful tool for social connection, fostering empathy and understanding through collective storytelling.

## Application of PT in the healthcare domain and beyond

PT has been effectively utilized to enhance communication and empathy in medical and allied health education. By engaging in PT, healthcare professionals can improve their communication skills, leading to a more compassionate and empathetic healthcare environment (Lipsker 2005, Salas *et al.* 2013, Brown *et al.* 2018). PT provides a creative and supportive space for exploring complex emotions, which can positively impact mental health. This supportive environment fosters a sense of agency and validation, crucial for personal and professional growth in healthcare settings (Jain 2019). Qualitative reports indicate that PT participants often experience enhanced self-esteem, greater self-knowledge, fun and relaxation, and an increased sense of connection and empathy towards others (Moran and Alon 2011). By focusing on the exploration of life stories through a “dramatic reality”, PT creates a unique platform for life-review principles, which have proven particularly beneficial when working with older adults (Keisari *et al.* 2018). PT offers a multifaceted approach to improving well-being by fostering empathy, enhancing communication, and supporting mental health recovery (Munjuluri *et al.* 2020). Needa (2018), a PT practitioner, reported that PT has been used in a wide variety of social settings. In the Northern Territories of Australia, Aboriginal communities used PT to enhance their self-esteem and to recover from substance abuse. In Argentina, people used PT to revisit their social and political history. In Kabul, PT enabled the sharing of untold stories in a court of law. In Washington DC, PT has been embedded in the school curriculum to promote the practice of inner listening. Other researchers used PT in studies related to trauma recovery, migration, gender-based violence, racism, and community healing (Fox 1994, Sajnani 2012, Salas 2013, Gonzalez *et al.*, 2024). These examples illustrate the potential of PT to bring lived experiences to the surface and stimulate dialogue around social issues. Thus, PT can be a valuable opportunity to deepen our understanding of health systems by employing a participatory, embodied, and narrative-based method.

## What can PT offer to HPSR storytelling?

PT can add value to HPSR storytelling in four significant ways. Firstly, PT can help in perspective-building by allowing researchers, policymakers, and community members to see health system issues through the lived experiences of others. PT creates a congenial environment for people to speak from their hearts and to share their realities as perceived by them through personal stories (Fox 1994). As stories materialize on stage in PT sessions, people gain perspective and relativization of the issue. A pertinent example illustrating how PT aids in building perspectives on health system-related issues is found in a study conducted at Baylor College of Medicine in Houston, TX, USA (Salas *et al.* 2013). In this study, PT was integrated into a first-year medical student elective titled ‘Compassion and the Art of Medicine’. The sessions provided a platform for students to share personal narratives related to their experiences in medical education, which were then enacted by trained actors. This process facilitated open discussions about the emotional challenges of medical training, such as stress, empathy, and communication barriers. The study concluded that PT served as a powerful tool to enhance communication among medical students, allowing them to reflect on and better understand the complexities of the healthcare system from multiple perspectives. In another example, PT was used as an entry tool to the world of nurses in a palliative centre and a geriatric centre, in an attempt to understand their needs through the stories they shared of their demanding jobs. The concerns shared by nurses during the Playback performance were further explored in more detail through sessions of Expressive Arts therapy in an endeavour to help the nurses to develop a well-rounded approach towards the different situations that they encounter and tools for self-awareness and self-care (Jain 2019).

Secondly, PT processes constantly remind us to respect the inherent worth of human beings, their wisdom and lived experiences. This emphasis on human dignity can support efforts to centre respect, compassion, and inclusivity in HPSR storytelling. The foundational premise of participation is that value is placed on the dignity that is inherent to every individual and community (Sen 1999, WHO 2008, UNDP 2016, Hickey and Mohan 2004). Therefore, participatory methods in principle enable the agency of participants ‘to speak up, to engage, to experience oneself and be experienced as a person with the right to express oneself and to have the expression valued by others’ (Abma *et al.* 2019). In that sense, PT can be one of the instruments for respectful engagement with actors in HPSR. Through inviting communities in vulnerable circumstances to be tellers in Playback performances, PT can ‘offer a home to their voices’ (Good 2003 as cited in Zhang 2024). For example, from a dignity perspective, PT has been used to connect groups from Tamil, Sinhala, and Muslim backgrounds in Sri Lanka. PT sessions in Sri Lanka provided spaces for sharing personal stories of conflict, loss, and resilience, which were immediately enacted by trained performers. Participatory strategies, such as co-designing with community leaders and multilingual facilitation, helped bridge linguistic and cultural divides. PT, by centring dignity, validated diverse experiences, fostered empathy, and promoted collective meaning-making. Outcomes included strengthened intergroup relationships, a heightened sense of collective humanity, and small but significant shifts toward covenantal pluralism, where communities recognised their interconnectedness while honouring the differences and dignity of each other (Buhler King *et al.* 2023). PT can offer valuable contributions in similar HPSR contexts.

Thirdly, PT can support capturing the emotional geographies of health policy processes. The politics of social processes such as health policy making and implementation are not only matters of power, economics, and rationality but also ‘emotive realities’ of how resources are mobilized, accessed, utilized, or disputed (Sultana 2011, Borén *et al.* 2021). The influence of emotions such as anger, fear, enthusiasm etc. and their impact on drawing attention to social problems, shaping perceptions of policy actors, encouraging political participation, and mobilizing advocacy coalitions for policy changes has been extensively examined in the literature (Pierce 2021). Though not expressly stated, these emotions are an integral part of theories and practices of (health) policy processes. PT allows individuals to narrate their personal stories, which often contain rich emotional details. These stories can reveal how people experience and navigate their internal and external worlds. The performance then translates these individual experiences into a collective understanding of emotional landscapes, highlighting common themes and individual variations (Fox 1994, Sajnani 2012, Salas 2013). In the context of health policy, this could mean understanding how different locations (e.g. healthcare facilities, communities, homes) and policies affect the emotional well-being of individuals and groups. By portraying personal stories, PT reveals commonalities among community members. Whether through themes of triumph, struggle, or everyday life, the enacted stories can illustrate shared experiences and concerns, helping individuals see themselves in the stories of others. The process of watching personal stories come to life helps community members empathise with each other's experiences (Ng and Graydon 2016). PT's emphasis on emotional expression and representation allows individuals to deeply engage with the stories of others, leading to greater empathy and understanding across diverse social groups. In HPSR, this capacity of PT to foster empathy and highlight shared emotional realities can deepen understanding of systemic health challenges and build solidarity among stakeholders (Boydell *et al.* 2012, George *et al.* 2015). Such emotional engagement is crucial for co-creating more inclusive and responsive health policies and systems (Cargo and Mercer 2008, George *et al.* 2015).

Finally, PT can support the process of collectivization in HPSR. Social transformation begins with this collective understanding of our everyday realities, because it helps communities to recognise shared experiences and systemic patterns and to envision change. Participatory and performative methods such as PT foster this understanding and collective agency (Boal 1995, Thompson 2003). The act of watching someone's story being performed can help the audience reduce anxiety, understand, and relate to the emotional geographies of others (Fox 1994, Salas 2013, Munjuluri *et al.* 2020). This shared experience can bridge gaps between different perspectives and create a deeper appreciation for the diversity of human experiences. By theatrically materializing the individual stories of the participants on the stage, collectively, the participants weave a deeper web of larger stories embedded in the societies in which they live. PT enables us to tap into our collective and universal experiences through storytelling (Fox 1994, Sajnani 2012, Salas 2013). One illustrative case is the ‘Storytelling for Change’ project conducted by the non-profit organization Tostan International (2014) in West Africa, which used community storytelling and performance methods similar to PT to address maternal and child health challenges. In these sessions, women shared personal experiences of accessing care during pregnancy, which were then dramatized by community facilitators. Stories of disrespect, lack of transportation, and unaffordable services surfaced, leading to the co-creation of health committees and influencing local health policies to improve maternal and child care access.

## How to operationalize PT in HPSR projects?

PT can be used during different stages of research to bring to the surface people's experiences with health systems, revealing the emotional realities behind statistics, such as barriers to health care, trust in providers, and health inequities that formal interviews, focus group discussions, or surveys might miss. By enacting the lived experiences of patients, health workers, or policymakers, PT can be used to foster empathetic conversations among diverse stakeholders, helping bridge power gaps and creating a more collaborative space for policy discussions. Instead of only using reports, policy briefs, or presentations, researchers can use PT performances to bring research findings to life, making complex issues more relatable and memorable for policymakers, practitioners, and the public. Finally, in post-conflict or under-resourced health settings, PT can be a tool for emotional healing, restoring trust, and supporting resilience among frontline workers, care-seeking individuals, and communities, laying a stronger foundation for future health system reforms.

PT can be operationalized as a participatory method in HPSR by engaging trained practitioner-researchers who possess both PT expertise and skills in critical dialogue facilitation (Fox 1994, Salas 2013). Participants and performers may include community members, frontline health workers, and policymakers, selected to reflect a diversity of lived experiences within the health system (Boydell *et al.* 2012). Recruitment can be achieved through community-based networks and purposive sampling strategies that emphasise trust and inclusivity. Systematic data collection using PT might involve video documentation of performances, field notes, and facilitated post-performance reflections, with informed consent processes that explicitly address ownership and ethical dissemination of performative data (Sajnani 2012). PT is rooted in the oral tradition and values the immediacy of live storytelling. However, selective recording can be conducted with informed consent and adherence to ethical guidelines. Like in any other participatory research practice, data analysis must account for the co-created and interpretive nature of PT and requires a reflexive, democratic approach to meaning-making (Cargo and Mercer 2008). When using PT in HPSR, it is important to prioritize the emotional safety of participants. If need be, participants should have access to psychosocial support, such as on-site counsellors or referrals, because some stories can be intense to process. Facilitators must be sensitive to power dynamics, ensuring marginalized voices are heard. Cultural adaptation is crucial to ensure alignment with local values and norms. Together, these precautions help create ethical, inclusive, and respectful storytelling spaces in HPSR projects using PT methodology.

## Anticipated challenges in using PT as a methodology in HPSR storytelling processes

Applying PT in HPSR storytelling without a grounding in critical consciousness (Freire 1970) (i.e. without the capacity to recognise, question, and act upon the social, political, and economic contradictions that shape lived experiences) can risk reproducing the very power dynamics and exclusions it aims to challenge. Without a deliberate, reflective engagement with issues of privilege, marginalization, and structural oppression, PT may become a superficial tool, one that merely showcases stories without interrogating the systems that shape them. This can lead to the tokenization of participants’ experiences, especially those from historically underrepresented or oppressed communities, reducing complex social realities to performative moments devoid of context or accountability. Moreover, without critical awareness, facilitators may inadvertently centre dominant narratives or fail to create genuinely safe and brave spaces for storytelling. This undermines the epistemological promise of PT as a method rooted in co-creation, social justice, and transformative dialogue (Freire 1970, Thompson 2003, Sajnani 2012). Although PT seeks to create a safe and welcoming space, not all participants may feel equally comfortable sharing their stories. Factors such as trauma, power imbalances, or cultural barriers can make some feel excluded.

It is also equally important to critically examine who is doing PT for whom and why (i.e. who is telling stories, whose stories are being told, whose stories are missing). Audience context shapes the meaning, impact, and inclusivity of PT in health policy and systems research. For example, a PT performance about access to healthcare might highlight systemic failures to an urban audience but focus on trust in community health workers in rural settings. The emotional impact shifts too: frontline workers may feel validated, while policymakers might feel challenged. Inclusivity depends on adapting language, references, and participation styles to different groups. For instance, it may be necessary to create homogeneous or heterogeneous participant groups, depending on the context and objectives. In short, PT in HPSR must respond to audience context, as it fundamentally transforms how messages are received and acted upon.

Performers and conductors should know the cultural nuances of the communities/actors to whom PT is performed and should use the language of the community. It is a moral and ethical imperative that communities themselves should be able to claim their agency through PT (i.e. PT performed by the actors of the communities they belong to after some initial support and capacity building for these communities on PT). This approach aligns with the participant performance model, which emphasises active involvement of the community members in the sharing and performance of the stories, rather than merely being passive subjects of external performances (Fox 2020). PT is meaningful when it is managed by a conductor and actors who are sensitive to the larger socio-political frame in which the stories of the participants are embedded. PT sessions can have an explicit theme where participants share stories concerning the theme. PT can also be performed without a theme to begin with, yet at the end, the patterns in the stories shared by the participants may reveal underlying concerns and common interests of the participants.

## Conclusion

Stories are a form of expressing human experiences. Effective storytelling has the power to evoke deep human emotions and connections. There are various verbal and non-verbal methods used for storytelling. PT is an improvizational and interactive performance theatre format where (citizen) actors use their awareness, spontaneity, creativity and theatrical skills to retell the participants’ story on the spot. If applied critically, we argue that PT methodology has the potential to illuminate HPSR storytelling processes, given its ontological and epistemological alignment with social constructivism and its orientation towards values such as human dignity and social justice. By fostering deep listening and empathy, PT helps researchers and practitioners connect emotionally with stakeholders, strengthening co-creation. PT not only enhances qualitative inquiry in HPSR but also supports the broader shift toward health systems that are relational, inclusive, and responsive.

## Data Availability

No new data were generated or analysed in this article.

## Reflexivity statement

The authors are women in their 30s and 40s from India. M.P. is a health system and health policy researcher who uses applied theatre methodologies in her research with Adivasi communities in Southern India. R.J. is an applied theatre practitioner with 12 years of experience in training, teaching, and writing about Playback Theatre. M.P. and R.J. share a common interest in promoting the use and accessibility of applied theatre methods to different arenas of life.

## References

[czaf038-B1] Abma T, Banks S, Cook T et al Participatory Research for Health and Social Well-Being. Switzerland: Springer, 2019.

[czaf038-B2] Boal A . Theatre of the Oppressed. London: Pluto Press, 1979.

[czaf038-B3] Boal A . The Rainbow of Desire: The Boal Method of Theatre and Therapy. London: New York: Routledge, 1995.

[czaf038-B4] Borén T, Grzyś P, Young C. Policymaking as an emotionally charged arena: the emotional geographies of urban cultural policymaking. Int J Cultural Policy 2021;27:449–62. 10.1080/10286632.2020.1792891

[czaf038-B5] Boydell KM, Gladstone BM, Volpe T, et al The production and dissemination of knowledge: a scoping review of arts-based health research. Forum Qual Soc Res. 2012;13:Article 32. 10.17169/fqs-13.1.1711

[czaf038-B6] Brown A, Ramsay N, Milo M et al How research-based theatre is a solution for community engagement and advocacy at regional medical campuses: the Health and Equity through Advocacy, Research, and Theatre (HEART) program. Can Med Educ J 2018;9:e6–13. 10.36834/cmej.42191 PMC6104337 30140330

[czaf038-B7] Bruner J . Acts of Meaning: Four Lectures on Mind and Culture. he Jerusalem-Harvard Lectures. Cambridge: Harvard University Press, 1990.

[czaf038-B8] Bruner J . The narrative construction of reality. Crit Inq 1991;18:1–22. 10.1086/448619

[czaf038-B9] Buhler King C, Sivagnanam RJ, De Silva S. A participatory arts application of Playback Theatre to transitional justice in Sri Lanka. Res Drama Educ 2023;28:126–42. 10.1080/13569783.2023.2170221

[czaf038-B10] Cairney P, Kwiatkowski R. How to communicate effectively with policymakers: combine insights from psychology and policy studies. Palgrave Commun 2017;3:37. 10.1057/s41599-017-0046-8

[czaf038-B11] Cargo M, Mercer SL. The value and challenges of participatory research: strengthening its practice. Annu Rev Public Health 2008;29:325–50. 10.1146/annurev.publhealth.29.091307.083824 18173388

[czaf038-B12] Dahlstrom MF . Using narratives and storytelling to communicate science with non-expert audiences. Proc Natl Acad Sci U S A 2014;111:13614–20. 10.1073/pnas.1320645111 25225368 PMC4183170

[czaf038-B13] Desmond C, Brubaker KA, Ellner AL. Decision-making strategies: ignored to the detriment of healthcare training and delivery? Health Psychol Behav Med 2013;1:59–70. 10.1080/21642850.2013.854706 25264501 PMC4164239

[czaf038-B14] Epstein D, Heidt J, Farina C. The value of words: narrative as evidence in policymaking. Evid Policy 2014;10:243–58. 10.1332/174426514X13990325021128

[czaf038-B15] Fadlallah R, El-Jardali F, Nomier M et al Using narratives to impact health policymaking: a systematic review. Health Res Policy Syst 2019;17:26. 10.1186/s12961-019-0423-4 30836972 PMC6402129

[czaf038-B16] Fiddian-Green A, Gubrium A. Critical narrative intervention for health equity research and practice: editorial commentary introducing the health promotion practice critical narrative intervention special collection. Health Promot Pract 2021;22:2S–7S. 10.1177/15248399211046185 34664521

[czaf038-B17] Fox J . Acts of Service: Spontaneity, Commitment, Tradition in the Non Scripted Theatre. New York: Tusitala Publishing, 1994.

[czaf038-B18] Fox J . Unexpected resilience of the participant performance model for playback theatre. In: Prentki T, Preston S (eds.) The Applied Theatre Reader. London: Routledge, 2020, 211–8.

[czaf038-B19] Fraser KD, al Sayah F. Arts-based methods in health research: a systematic review of the literature. Arts Health 2011;3:110–45. 10.1080/17533015.2011.561357

[czaf038-B20] Freire P . Pedagogy of the Oppressed. London: Continuum, 1970.

[czaf038-B21] Friel S, Townsend B, Fisher M et al Power and the people’s health. Soc Sci Med 2021;282:114173. 10.1016/j.socscimed.2021.114173 34192622

[czaf038-B22] George AS, Mehra V, Scott K et al Community participation in health systems research: a systematic review assessing the state of research, the nature of interventions involved and the features of engagement with communities. PLoS One 2015;10:e0141091. 10.1371/journal.pone.0141091 26496124 PMC4619861

[czaf038-B23] Gonzalez A-J, de Lima PM, Preto L et al Playback theatre applications: a systematic review of literature. Arts Psychother 2024;89:102152. 10.1016/j.aip.2024.102152

[czaf038-B24] Gore R, Parker R. Analysing power and politics in health policies and systems. Glob Public Health 2019;14:481–8. 10.1080/17441692.2019.1575446 30773135

[czaf038-B25] Greenhalgh T, Jackson C, Shaw S et al Achieving research impact through co-creation in community-based health services: literature review and case study. Milbank Q 2016;94:392–429. 10.1111/1468-0009.12197 27265562 PMC4911728

[czaf038-B26] Gubrium A, Turner KN. Digital storytelling as an emergent method for social research and practice. In: Hesse-Biber SN (ed.) Handbook of Emergent Technologies in Social Research. New York: Oxford University Press, 2011, 469–91.

[czaf038-B27] Hallward P . Staging equality: Rancière’s theatrocracy. New Left Rev 2006;37:109–29. 10.1515/9781478090977-011

[czaf038-B28] Hickey S, Mohan G. Participation: From Tyranny to Transformation? Exploring New Approaches to Participation in Development. London: Zed Books Ltd, 2004.

[czaf038-B29] Hooks B . Feminism is for Everybody: Passionate Politics. Cambridge, MA: South End Press, 2000.

[czaf038-B30] Jain R . Playback theatre as an entry point tool to understand concerns of caregivers in the health care sector in India. In: Gopal B, Tasker B (ed.) Playback Theatre Around the World: Diversity of Application. Bangalore: CHRIST (Deemed to be University), 2019, 1–8.

[czaf038-B31] Jeevika . Jeeta Vimukti Karnataka. 2025. https://jeevikafree.org/ (12 May 2025, date last accessed).

[czaf038-B32] Jewell CJ, Bero LA. “Developing good taste in evidence”: facilitators of and hindrances to evidence-informed health policymaking in state government. Milbank Q 2008;86:177–208. 10.1111/j.1468-0009.2008.00519.x 18522611 PMC2690362

[czaf038-B33] Jones MD, McBeth MK. A narrative policy framework: clear enough to be wrong? Policy Stud J 2010;38:329–53. 10.1111/j.1541-0072.2010.00364.x

[czaf038-B34] Keisari S, Yaniv D, Palgi Y et al Conducting playback theatre with older adults: a therapist’s perspective. Arts Psychother 2018;60:72–81. 10.1016/j.aip.2018.07.002

[czaf038-B35] Lipsker C . Constructing Nursing Knowledge Via Playback Theatre in Professional Training of Community Nurses: A Study of the Effect on Learning and Behaviour Patterns. Doctoral thesis, University of Haifa, Faculty of Education, Department of Education, 2005.

[czaf038-B36] Lorenz D . Participatory visual and digital methods by Aline Gubrium & Krista Harper. Alberta J Educ Res 2015;60:748–50. 10.11575/ajer.v60i4.55849

[czaf038-B37] Moran GS, Alon U. Playback theatre and recovery in mental health: preliminary evidence. Arts Psychother 2011;38:318–24. 10.1016/j.aip.2011.09.002

[czaf038-B38] Mullen M, Freebody K. Rethinking the relationship between applied theatre and policy. Res Drama Educ 2022;27:271–85. 10.1080/13569783.2022.2085032

[czaf038-B39] Munjuluri S, Bolin PK, Amy Lin YT et al A pilot study on Playback Theatre as a therapeutic aid after natural disasters: effects on PTSD, anxiety, and resting-state connectivity. Chronic Stress (Thousand Oaks) 2020;4:2470547020966561. 10.1177/2470547020966561 33210057 PMC7643224

[czaf038-B40] Mwisongo A, Nabyonga-Orem J, Yao T et al The role of power in health policy dialogues: lessons from African countries. BMC Health Serv Res 2016;16:213. 10.1186/s12913-016-1456-9 27454227 PMC4959373

[czaf038-B41] Needa V . *An Introduction to Playback Theatre,* 2018. https://trueheart.org.uk/wp-content/uploads/2013/05/INTRODUCING-PLAYBACK-THEATRE-Sept-2018-.pdf (4 December 2024, date last accessed).

[czaf038-B42] Ng SHL . Cultivating the artistry of citizen actor: aesthetics, pedagogy and training exercises for Playback Theatre as art. In: Playback Theatre Practice: Selected Articles. Moscow: LLC Vash Poligraphichesky Partner Publishing House, 2015, 212–28.

[czaf038-B43] Ng HC, Graydon C. In search of empathy in Playback Theatre: a preliminary study. Person-Centred Exp Psychother 2016;15:126–41. 10.1080/14779757.2016.1172252

[czaf038-B44] Nicholson H . Applied Drama: The Gift of Theatre. England: New York: Palgrave Macmillan, 2005.

[czaf038-B45] Nisker J, Martin DK, Bluhm R et al Theatre as a public engagement tool for health-policy development. Health Policy 2006;78:258–71. 10.1016/j.healthpol.2005.10.009 16337306

[czaf038-B46] O’Connor P, O’Connor B. Research in drama education. Res Drama Educ 2009;14:471–7. 10.1080/13569780903285966

[czaf038-B47] Pierce JJ . Emotions and the policy process: enthusiasm, anger and fear. Policy Polit 2021;49:595–614. 10.1332/030557321X16304447582668

[czaf038-B48] Prentki T, Preston S (eds.). The Applied Theatre Reader. London: New York: Routledge, 2009.

[czaf038-B49] Rancière J . Hatred of Democracy. London: New York: Verso, 2006a.

[czaf038-B50] Rancière J . The ethical turn of aesthetics and politics. Critical Horizons 2006b;7:1–20. 10.1163/156851606779308242

[czaf038-B51] Rancière J . Contemporary art and the politics of aesthetics. In: Hinderliter B, Kaizen W, Maimon V, Mansoor J, McCormick S (eds.) Communities of Sense: Rethinking Aesthetics and Politics. Durham, NC: Duke University Press, 2009, 31–50.

[czaf038-B52] Sajnani N . Response/ability: towards a critical race feminist approach to drama therapy. Arts Psychother 2012;39:186–92. 10.1016/j.aip.2011.12.009

[czaf038-B53] Sajnani N, Johnson DR. Opening up Playback Theatre: perspectives from developmental transformations and the theatre of the oppressed. Chest Broken Toys J Dev Transform 2016;1:94–126. https://playbacktheatre.org/playbacktheatre/wp-content/uploads/2011/03/Opening-Up-Playback-Theatre-.pdf (12 May 2025, date last accessed).

[czaf038-B54] Salas J . Stories in the moment: Playback Theatre for building community and justice. In: Program for Peacebuilding and the Arts, International Center for Ethics, Justice and Public Life, Acting Together on the World Stage: Performance and the Creative Transformation of Conflict, Vol. 2, Chapter 4. Oakland, CA: Brandeis University, 2011, 93–123.

[czaf038-B55] Salas J . Improvising Real Life: Personal Story in Playback Theatre. New York: Tusitala Publishing, 2013.

[czaf038-B56] Salas R, Steele K, Lin A et al Playback theatre as a tool to enhance communication in medical education. Med Educ Online 2013;23:22622. 10.3402/meo.v18i0.22622 PMC3873757 24369762

[czaf038-B57] Sen A . Development as Freedom. Oxford: Oxford University Press, 1999.

[czaf038-B58] Sheikh K, Gilson L, Agyepong IA et al Building the field of health policy and systems research: framing the questions. PLoS Med 2011;8:e1001073. 10.1371/journal.pmed.1001073 21857809 PMC3156683

[czaf038-B59] Steiner JF . The use of stories in clinical research and health policy. J Am Med Assoc 2005;294:2901–4. 10.1001/jama.294.22.2901 16352799

[czaf038-B60] Sultana F . Suffering for water, suffering from water: emotional geographies of resource access, control and conflict. Geoforum 2011;42:163–72. 10.1016/j.geoforum.2010.12.002

[czaf038-B61] Thompson J . Applied Theatre: Bewilderment and Beyond. Oxford, UK: Peter Lang Ltd, 2003.

[czaf038-B62] Toolan M . Narrative: A Critical Linguistic Introduction. London: Routledge, 2001.

[czaf038-B63] Tostan International . *Using Storytelling to Reduce Child Mortality in Senegal*. Tostan International, 2014. https://tostan.org/using-story-telling-reduce-child-mortality-senegal/ (4 June 2025, date last accessed).

[czaf038-B64] United Nations Development Programme (UNDP) . Human Development Report 2016: Human Development for Everyone. New York: UNDP, 2016.

[czaf038-B65] Vuuren VJ, Bjorn R, Ayanda K. Theatre and Democracy: Building Democracy in Post War & Post Democratic Contexts. Oslo, Norway: Cappelen Damm Akademisk/NOASP, 2021.

[czaf038-B66] World Health Organization . Closing the Gap in a Generation: Health Equity Through Action on the Social Determinants of Health. Geneva: WHO Commission on Social Determinants of Health, 2008. 10.1016/S0140-6736(08)61690-6 18994664

[czaf038-B67] Zhang M . Beyond the entertainment purposes of theatre: playback theatre and its implications in a social context. Scholarly Rev J. 2024;2024:1–8. 10.70121/001c.123641

